# Relationship between maximal respiratory pressures and multiple childbearing in Brazilian middle-aged and older women: A cross-sectional community-based study

**DOI:** 10.1371/journal.pone.0208500

**Published:** 2018-12-04

**Authors:** Ingrid Guerra Azevedo, Saionara Maria Aires da Câmara, Catherine McLean Pirkle, Álvaro Campos Cavalcanti Maciel, Elizabel de Souza Ramalho Viana

**Affiliations:** 1 Physiotherapy Department, Universidade Federal do Rio Grande do Norte, Natal Rio Grande do Norte, Brazil; 2 Faculdade de Ciências da Saúde do Trairi, Universidade Federal do Rio Grande do Norte, Santa Cruz, Rio Grande do Norte, Brazil; 3 Office of Public Health Studies, University of Hawaiʻi at Mānoa, Honolulu, Hawaiʻi, United States of America; Monash University, AUSTRALIA

## Abstract

**Objective:**

Previous studies show that multiparity and a number of chronic conditions are correlated among women. Also, low respiratory muscle strength has been associated to adverse health outcomes such as chronic lung disease and early mortality. This study aimed to investigate associations between the number of lifetime pregnancies and maximal inspiratory/expiratory pressures.

**Methods:**

In a cross-sectional study, 204 women ages 41–80 years-old, from the rural community of Santa Cruz, Brazil, provided data regarding demographics, socioeconomic characteristics, health behaviors, and number of lifetime pregnancies (≤3, 4–6 or ≥7). Maximal respiratory pressures were measured with a digital manometer. Multiple linear regression analysis was used to examine the association of multiple childbearing on maximal respiratory pressures.

**Results:**

Of the participants, 44.1% had ≤3 pregnancies, 30.4% had 4–6 pregnancies and 25.5% had >7 pregnancies. In the unadjusted analyses, maximal inspiratory and expiratory pressures varied significantly according to multiple childbearing categories. After adjustment, the values remained statistically significant only for maximal expiratory pressure. Compared to women with ≤3 lifetime pregnancies, those who had ≥7 pregnancies had significantly lower maximal expiratory pressure values (β = -18.07, p = 0.01)

**Conclusion:**

Multiple childbearing appears to be negatively associated with maximal respiratory pressures; women with a higher number of lifetime pregnancies had lower values of maximal respiratory pressures when compared to those with fewer pregnancies. This association may be due to biomechanical changes in the respiratory muscles promoted by multiple lifetime pregnancies. This finding indicates a need to motivate women, from the prenatal to postpartum period, to safely exercise their respiratory muscles, including abdominal muscle exercises as well as respiratory muscle training.

## Introduction

Aging is a dynamic and progressive process, influenced by physiological, socioeconomic, and biological factors [1,2]. Among physiological factors linked to aging, numerous alterations in the respiratory system negatively impact respiratory volumes and capacities [3], contributing to the greater burden of morbidities commonly observed in this population [4].

Aging-associated alterations in the respiratory system that contribute to respiratory muscle strength decline may include pulmonary volumetric and thoracic changes [4,5]. For example, research shows a reduction in muscle mass and strength with age [6,7], which consequently weakens peripheral (limbs) and respiratory muscles. Vast literature indicates a reduction of peripheral muscle strength with increasing chronological age [8,9]. Moreover, compared to younger adults, older adults show a reduction of up to 25% in their diaphragmatic strength [7] and some studies point to age as predictive for maximal respiratory pressures values, based on evidence that the older the subject, the lower the pressures [10–13]; although there is also evidence that shows small [14,15] or no relationship between MIP/MEP and age in older subjects [16], in older adults, some evidence also points to an association between respiratory muscle strength and physical function [17,18], as measured with lower limb speed tests [19].

Declining respiratory muscle strength is associated with mobility decline in older persons, independent of lower extremity strength and physical activity [18,20]. Additionally, other variables, as female sex and socioeconomic adversity contribute to reduced physical function in older adults [2,21,22]. However, these associations are typically assessed in peripheral muscles, such as in the legs. Studies about inspiratory (diaphragm, external intercostals and accessory muscles) and expiratory muscle (abdominals) strength that take into consideration aspects related to socioeconomic status and sex are scarce in the literature. More research is needed to explore potential associations between social risk factors and respiratory function.

Based on previous robust evidence of associations between socioeconomic measures and physical function assessed in the peripheral muscles [2,21,22] and a small amount of evidence of female-specific risks (e.g. reproductive variables), we would expect associations between female reproductive, socioeconomic and respiratory measures. Yet, this topic has been less investigated in the literature when considering respiratory muscles strength.

To our knowledge, only one study has investigated the association between the aforementioned factors and respiratory outcomes, and it found that multiparity is associated with a decrease in respiratory muscle strength, mainly among women ages 60 years and over [23]. However, these authors only investigated maximal inspiratory pressure (MIP) and did not examine maximal expiratory pressure (MEP). They found that the higher the parity of the woman, the weaker the MIP, and the authors attributed the findings to anatomical and hormonal changes related to multiple childbirths that may impair muscle contraction, and, consequently, reduce MIP [23]. Examination of MEP would allow for the evaluation of the strength of the expiratory muscles, which participate in the mechanism of breathing, assist intestinal transit, and clean the airways through coughing [24]. If there is indeed an association between having multiple childbirths and respiratory outcomes later in life, then examination of both MIP and MEP would provide a more complete picture of the possible physiological mechanisms that might relate these two factors (e.g. childbearing and later life respiratory function).

Female reproductive history indicators such as age-at-first-childbirth and the effects of menopause appear to contribute to the physical alterations that coincide with aging. Such alterations include earlier declines in female ventilator functional capacity, in comparison to men at similar ages [25,26]. Studies indicate that women with early childbearing and/or 3 or more childbirths present worse physical performance, measured primarily through tests of the lower limbs (gait speed and chair stand tests), in comparison to women who had children after 20 years of age and fewer children [25–27].

Some studies have observed associations between female reproductive events such as early-maternal-age-at-first-birth and chronic lung disease [25,28], but only reported the results as surprising findings with little investigation into the potential reasons for the noted associations. Thus, there is a need for more research to corroborate these findings. There is also a need for more research about the implications of socioeconomic and lifestyle factors, which likely have an association with both female reproductive histories and respiratory function later in life.

This study investigates whether there are associations between female reproductive history indicators, respiratory muscle strength, and socioeconomic factors (such as income and educational levels) in women from a rural community in Northeast Brazil. Such a study is important to better understand factors that may contribute to loss of respiratory function across the life-course in women, given that preservation of respiratory muscle strength in women who have had many children throughout their lives is important to reduce the effects of the anatomical and hormonal changes related to multiple childbearing, particularly when associated with the changes resulting from the process of aging.

## Material and methods

### Population and sample

The study sample was composed of women ages 41–80 years old (N = 204) living in the rural community of Santa Cruz, which is located 120 km from Natal, the capital of the state of Rio Grande do Norte (RN), Brazil. This community has a population of roughly 36,000 inhabitants over a landmass of approximately 624,356 km^2^. This community is relatively homogeneous, with most of the population considered lower-middle class. Santa Cruz ranks 3,393 of 5,565 Brazilian municipalities with Human Development Index (HDI) scores. The HDI is a composite index of life expectancy, years of schooling and income [29], which serves as a comparison index for different populations. According to the latest census data [30] from 2010, Santa Cruz has an HDI of 0.635, which is considered medium in the HDI classification system [29]; approximately 60% of the population is considered vulnerable to poverty [30], which means “those who are below or at risk of falling below a certain minimally acceptable threshold of critical choices across several dimensions, such as health, education, material resources, security” [29].

In this cross-sectional study, we aimed to sample 50 women for each of the following age groups: 41–50; 51–60; 61–70; 71–80 years old. This sampling was done to ensure a relatively equal representation of participants across the age range of interest. Without deliberate sampling of older age categories, the sample would have been skewed to the younger categories, as it was easier for younger women to travel to the evaluation site in central Santa Cruz.

A total of 208 women were recruited by advertisements in primary health care units and community centers across the city, according to the following two inclusion criteria: aged 41–80 years and living in Santa Cruz. We excluded women with neurological impairments, degenerative diseases, or any chronic condition that could compromise the respiratory muscle strength evaluation. Women with severe cognitive impairment, defined as four or more errors on the orientation scale of the Leganes Cognitive Test [31], were also excluded, as this is considered an indication of inability to complete the study protocols. Four presumably eligible women were also excluded because they could not follow the instructions for developing a maximal respiratory pressure per requirement. Thus, 204 women comprised our final sample.

### Procedures & measures

All participants were evaluated by trained interviewers at the Faculty of Health Sciences of Trairi, a satellite campus of the Federal University of Rio Grande do Norte located in Santa Cruz.

### Ethics

All participants were informed of the objectives and procedures of the research study at first contact and signed a consent form. The study protocol received ethics approval by the Ethics and Research Committee of the Federal University of Rio Grande do Norte.

### Outcome measures

#### Maximal respiratory pressure

Respiratory muscle strength was assessed with an electronic manometer MVD 300 (-300 a +300 cmH_2_0) (Globalmed, Porto Alegre, Rio Grande do Sul, Brazil), supervised by a trained interviewer. Individuals were stationed in a sitting position and asked to perform a maximal expiration (near to the residual volume) followed by a maximal inspiration (near to total lung capacity) for the maximal inspiratory pressure (MIP) assessment. A maximal inhalation (near to total lung capacity) followed by a forced expiration was performed for the maximal expiratory pressure (MEP) assessment. The highest value obtained in up to five attempts was chosen for MIP and MEP, since none of the participants’ values of the best three trials differed by more than 10% from each other. A one minute of interval was allowed between the attempts; a flanged mouthpiece was used for the tests [32].

#### Reproductive history

Two variables were self-reported: 1) maternal age at first childbirth and 2) number of pregnancies. Lifetime childbearing was categorized as three pregnancies or fewer, four to six pregnancies, or seven pregnancies or more. This variable was created based on the literature related to lifetime childbearing. The first category was based on previous studies indicating that women with three or more children have worse physical performance and a greater chance of developing chronic diseases [24,25]. Since 25% of our sample reported seven or more lifetime pregnancies, we used this cutoff to create the third category [33].

In order to categorize whether the woman had her first child as an adolescent or adult, the age when she first gave birth was divided into less than 18-years-old and 18- years or more. If she did not have children, she was considered nulliparous [26]. Eighteen years is the legal age of majority in Brazil.

#### Potential confounders

Age: age was considered a covariate in these analyses because it is related to respiratory muscle strength and to childbirth [3,10–16,23].

Body mass index (BMI): multiparity is associated with greater body weight [26,34]. BMI has also been associated with both higher and lower respiratory muscle strength [35,36]. Measures of height (m) and weight (kg) were taken using a scale with a stadiometer (WELMY, W100H, model R-110, Santa Bárbara d'Oeste, Brazil) to calculate the BMI (kg/m^2^).

Socioeconomic position: family income and education were considered potential confounders since both are associated with reproductive history and respiratory muscle strength [20, 25, 37]. Income and educational data were also self-reported. Income was categorized using the Brazilian minimum monthly wage (MW) as a reference, which is defined as the lowest remuneration that workers can receive as payment for their jobs per month. The definition of minimum wage states that it is an amount that should be able to supply the normal basic needs for a family. At the time of the interview, the MW was equal to R$880.00 (around USD$250.00) per month. Nevertheless, according to the Statistics and Socioeconomic Studies Department of Brazil (DIEESE) [38], the sufficient minimum salary to meet this definition, at the time of data collection, should be more than four times the value established by the Brazilian government. In our study, family income was thus dichotomized as less than 3MW and 3MW or more, as a way to distinguish those who are below this amount [37]. Educational data were categorized into less than seven years (less than basic education) or seven or more years (basic education or more) [37].

Physical activity: physical activity measures are associated with both reproductive history and respiratory measures [21,23]. To assess physical activity, we asked the participants how many days and how many times per day they had walked continuously for 10+ minutes during the last week. This measure included walking as a way to go from one place to another, or as physical exercise. Subsequently, participants were categorized into one of two categories: less than 90 min/week and 90 min/week or more [37]. To evaluate the time per day spent in sedentary activity, women were asked to report how much time in a normal day they remained seated, and the variable was dichotomized at 4 hours/day or less, and more than 4 hours/day [37].

Smoking: women with a high lifetime number of pregnancies are more likely to be smokers [23], and tobacco use is associated with respiratory muscle strength [39]. We asked participants about their habits in relation to smoking (current smoker, previous smoker or never smoker). Since few women reported being current smokers (N = 13; 6.4%), we dichotomized the women into two groups: ever smoked and never smoked.

### Statistical analysis

Data analyses were conducted using SPSS software, version 20.0 (SPSS, Chicago, IL, USA). Descriptive statistics for all variables were presented according to the variable pregnancies and analyzed using analysis of variance (ANOVA) and post hoc Tukey tests for continuous variables. For categorical variables, Chi-square tests were used for comparison of proportions. Means and standard deviations of MIP and MEP were presented for each category of the independent variables and compared using t-tests and ANOVA.

Multiple linear regression analyses evaluated the effect of multiple childbearing, e.g. number of reported lifetime pregnancies, on MIP and MEP adjusted for potential confounders. Three models were performed to evaluate the association of the variables of respiratory muscle strength and lifetime pregnancies after controlling for each group of covariates. In model 1, the associations among pregnancies and the outcome variables were adjusted for current age and age-at-first-birth. In model 2, the variables related to socioeconomic position (education and family income) were added. In model 3, the variables associated with lifestyle (smoking, sitting time per day and walking) and BMI were included. We centered the variables of age (participant’s age minus the minimum age in the sample, 41) and BMI (participant’s BMI minus the sample mean BMI, 29.04 kg/m^2^). Regression diagnostics were conducted for all linear analyses, including tests of collinearity, and no major deviations were noted.

## Results

Sample characteristics are presented according to lifetime pregnancy categories (Table 1). There was a significant difference in mean age across the groups: women with seven or more lifetime pregnancies were older than the women from the other groups, and women with four to six pregnancies were older than those who had fewer than three pregnancies. Educational attainment and income differed significantly across lifetime pregnancy categories. Greater proportions of women with lower levels of education and income were observed for the groups with higher numbers of lifetime childbearing. Compared to women with three or fewer pregnancies, those with seven or more pregnancies were marginally more likely to have ever smoked (38% versus 54%, p = 0.09). In contrast, those reporting seven or more pregnancies were significantly less sedentary than the lower pregnancy groups. Finally, for the age-at-first-birth variable, there was a gradient for the proportion of women reporting early first childbirth (before 18-years-old) across pregnancy categories; the proportion increased significantly with greater number of pregnancies (from 11% to 54%).

**Table 1:** Participant characteristics according to childbearing categories (N = 204).

Sociodemographic Data and Main Outcomes	Childbearing			
	3 or fewer	4 to 6	7 or more	p-value
	N (%)/ Mean (SD)			
**Age, y**	55.6 (10.6)	59.6 (10.4)	68.7 (8.6)	<0.01^a^
**Education, y**				<0.01^b^
Less than basic education (0–7 y)	22 (24.4)	40 (64.5)	47 (90.4)	
Basic education or more (≥ 8 y)	68 (75.6)	22 (35.5)	5 (9.6)	
**Income, MW**				0.02^b^
<3	56 (62.2)	49 (79.0)	42 (80.8)	
≥3	34 (37.8)	13 (21.0)	10 (19.2)	
**BMI, kg/m**^**2**^	29.60 (4.6)	29.43 (4.7)	27.57 (4.0)	0.02^c^
**Smoking**				0.09^b^
Ever	34 (37.8)	22 (35.5)	28 (53.8)	
Never	56 (62.2)	40 (64.5)	24 (46.2)	
**Walking, min/week**				0.25^b^
<90	85 (94.4)	54 (87.1)	46 (88.5)	
≥90	5 (5.6)	8 (12.9)	6 (11.5)	
**Sitting time per day, h**				<0.01^b^
4 or less	54 (60.0)	26 (41.9)	37 (71.2)	
>4	36 (40.0)	36 (58.1)	15 (28.8)	
**Age at first child, y**				<0.01^b^
Nulliparous	11 (12.2)	0 (0.0)	0 (0.0)	
< 18	10 (11.1)	19 (30.6)	28 (53.8)	
≥ 18	69 (76.7)	43 (69.4)	24 (46.2)	
Total	90 (44.1)	62 (30.4)	52 (25.5)	

MW: Minimum wage; BMI: Body Mass Index; Min: Minute; SD: standard deviation; h: hour; y: years; min/week: minute per week; kg/m^2^: kilogram per square meter

a: p value for ANOVA: 3 or fewer ≠ 4 to 6 ≠ 7 or more

b: Chi-square test

c: p value for ANOVA: 3 or fewer ≠ 7 or more

Table 2 displays unadjusted analyses for MIP and MEP values according to the study covariates. Mean MIP values differed significantly across education, smoking, sitting time per day and pregnancy categories. The mean MIP among those with less than three lifetime pregnancies was approximately 21 cm H_2_O greater than those with seven or more pregnancies. A similar difference (around 20 cm H_2_O) was observed for those with greater levels of education compared to lower educational attainment. When considering MEP values, there were significant differences according to education and lifetime pregnancies. As for MIP, those with three or fewer pregnancies had an average of 21 cm H_2_O more than those with seven or more pregnancies. The difference between educational groups was not as great as for MIP (12 cm H_2_O), but in the same direction.

**Table 2:** Means of MIP and MEP according to participant characteristics (N = 204).

Variables	MIP, cmH_2_O (mean, SD) (mean, SD)	p-value	MEP, cmH_2_O (mean, SD) (mean, SD)	p-value
**Education, y**		<0.01		<0.01
Less than basic education (0–7 y)	98.5 (41.9)		92.1 (30.7)	
Basic education or more (≥ 8 y)	118.4 (50.2)		104.9 (29.2)	
**Income, MW**		0.39		0.14
<3	106.0 (43.6)		96.1 (30.7)	
≥3	112.3 (54.8)		103.2 (30.0)	
**Smoking**		0.02		0.09
Ever	98.6 (45.1)		93.8 (27.8)	
Never	114.2 (47.3)		101.0 (32.2)	
**Walking, min/week**		0.87		0.32
<90	107.9 (46.8)		98.7 (30.9)	
≥90	106.1 (48.9)		91.4 (27.0)	
**Sitting time per day, h**		<0.01		0.49
4 or less	115.5 (49.8)		99.3 (27.7)	
>4	97.3 (40.7)		96.3 (34.2)	
**Age at first child, y**		0.76		0.97
Nulliparous	97.6 (51.8)		99.9 (31.4)	
< 18	108.7 (53.8)		98.4 (30.9)	
≥ 18	108.2 (43.6)		97.7 (30.6)	
**Number of lifetime pregnancies**		0.03^a^		<0.001^b^
3 or fewer	115.5 (48.9)		107.2 (30.0)	
4 to 6	107.9 (42.9)		94.4 (28.8)	
7 or more	94.2 (45.6)		86.5 (29.4)	

MW: Minimum wage; BMI: Body Mass Index; MIP: Maximal Inspiratory Pressure; MEP: Maximal Expiratory Pressure; h: hour; y: year; min/week: minute per week; cmH_2_O: centimeter of water

a: 3 or fewer > 7 or more

b: 3 or fewer > 4 to 6 > 7 or more

Table 3 shows the multiple linear regression results for MIP outcome. The reproductive history variables were not associated with MIP, after controlling for the covariates. Increasing age was significantly associated with lower mean MIP values in models 1 and 2, but not in model 3, which included BMI and health behaviors. In model 3, those who reported never smoking and limited sedentary behavior had significantly higher mean MIP values.

**Table 3:** Linear regression models to assess the relationship between reproductive history characteristics and MIP (N = 204).

	Model 1		Model 2		Model 3	
Variables	β (CI 95%)	p value	β (CI 95%)	p value	β (CI 95%)	p value
Intercept	134.74 (117.08 : 152.40)	<0.001	135. 83 (115.55 : 156.12)	<0.001	106.55 (74.96 : 138.14)	<0.001
**Number of lifetime pregnancies**						
7 or more	-12.32 (-32.52 : 7.88)	0.23	-9.52 (-30.85 : 11.80)	0.38	-7.18 (-28.11 : 13.74)	0.50
4 to 6	-6.06 (-21.99 : 9.87)	0.45	-3.82 (-20.65 : 13.00)	0.65	-0.34 (-16.81 : 16.13)	0.97
3 or fewer	0		0		0	
**Age at first child, y**						
Nulliparous	-15.78 (-48.72 : 17.16)	0.34	-16.18 (-49.34 : 16.96)	0.33	-14. 07 (-45.95 : 17.80)	0.38
≥ 18	-4.63 (-20.16 : 10.90)	0.55	-5.09 (-20.70 : 10.53)	0.52	-2.41 (-17.47 : 12.64)	0.75
< 18	0		0		0	
**Age, y**	-0.94 (-1.59 : -0.28)	<0.01	-0.83 (-1.56 : -0.09)	0.03	-0.60 (-1.32 : 0.11)	0.10
**Education, y**						
0–7			-6.41 (-24.60 : 11.78)	0.48	-6.98 (-24.43 : 10.47)	0.43
≥ 8			0			
**Income, MW**						
<3			-1.21 (-16.93 : 14.50)	0.88	0.02 (-15.29: 15.33)	0.99
≥3			0		0	
**BMI, Kg/m2**					2.05 (0.69 : 3.41)	<0.01
**Smoking**						
Never					13.38 (0.41: 26.33)	0.04
Ever					0	
**Walking, min/week**						
<90					1.16 (-20.01 : 22.34)	0.91
≥90					0	
**Sitting time per day, h**						
4 or less					20.90 (8.18 : 33.62)	<0.01
>4					0	

BMI: Body mass index; MW: Minimum wage; h: hour; y: year; min/week: minute per week; kg/m2: kilogram per square meter; CI: Confidence interval. Age was centered by subtracting the minimum age of the sample from the participant’s actual age. BMI was centered by subtracting the sample’s BMI mean from the participant’s actual BMI.

Table 4 shows the results of the multiple linear regression for MEP. There was a significant relationship between lifetime pregnancies and MEP, even in the fully adjusted model. Compared to women with three or fewer lifetime pregnancies, those with four to six pregnancies and seven or more pregnancies had significantly lower mean MEP values (-12.15 cm H_2_O and -18.07 cm H_2_O, respectively). No significant relationship was observed between MEP and age-at-first-childbirth. For both MIP and MEP, a greater mean BMI was associated with significantly greater values.

**Table 4:** Linear regression models to assess the relationship between reproductive history characteristics and MEP (N = 204).

	Model 1		Model 2		Model 3	
Variables	β (CI 95%)	p value	β (CI 95%)	p value	β (CI 95%)	p value
Intercept	118.13 (106.72 : 129.54)	<0.001	120.46 (107.36 : 133.57)	<0.001	107.43 (86.30 : 128.57)	<0.001
**Number of lifetime pregnancies**						
7 or more	-21.81 (-34.86 : -8.75)	<0.01	-20.81 (-34.59 : -7.03)	<0.01	-18.07 (-32.07 : -4.07)	0.01
4 to 6	-14.07 (-24.36 : -3.78)	<0.01	-13.20 (-24.07 : -2.33)	0.02	-12.15 (-23.17 : -1.13)	0.03
3 or fewer	0		0		0	
**Age at first child, y**						
Nulliparous	-13.12 (-34.40 : 8.15)	0.22	-12.83 (-34.25 : 8.59)	0.24	-10.88 (-32.20 : 10.44)	0.31
≥ 18	-7.84 (-17.87 : 2.20)	0.12	-7.99 (-18.09 : 2.10)	0.12	-6.57 (-16.64 : 3.50)	0.20
< 18	0		0		0	
**Age**	-0.22 (-0.65: 0.20)	0.29	-0.22 (-0.69 : 0.26)	0.37	-0.16 (-0.64 : 0.32)	0.51
**Education, y**						
0–7			-0.71 (-12.47 : 11.04)	0.90	-0.74 (-12.41 : 10.94)	0.90
≥ 8			0		0	
**Income, MW**						
<3			-3.55 (-13.71 : 6.59	0.49	-2.60 (-12.85 : 7.64)	0.62
≥3			0		0	
**BMI, kg/m**^**2**^					1.12 (0.20 : 2.03)	0.02
**Smoking**						
Never					4.87 (-3.80 : 13.54)	0.27
Ever					0	
**Walking, min/week**						
<90					4.81 (-9.35 : 18.99)	0.50
≥90					0	
**Sitting time per day, h**						
4 or less					3.55 (-4.96 : 12.06)	0.41
>4					0	

BMI: Body mass index; MW: Minimum wage; h: hour; y: year; min/week: minute per week; kg/m^2^: kilogram per square meter; CI: Confidence interval. Age was centered by subtracting the minimum age of the sample from the participant’s actual age. BMI was centered by subtracting the sample’s BMI mean from the participant’s actual BMI.

## Discussion

The results of this study suggest that multiple childbearing is associated with respiratory muscle strength, especially MEP. The association of multiparity and MEP was maintained even after extensive adjustment for covariates in the linear regression models. Overall, women with a greater number of lifetime pregnancies performed worse on the evaluation of respiratory pressures than women reporting fewer pregnancies.

It is important to note that women from the group with a higher number of lifetime pregnancies were significantly older than the other two groups. This is possibly because knowledge and opportunities for contraception have increased in Brazil over the past decades [40]. According to the literature [10], each additional year of age reduces MEP around 0.62 cmH_2_O in the Brazilian female population [10]. Since the mean difference of age between the groups with 3 or less and 7 or more lifetime pregnancies is 13.1 years, the expected difference in MEP related to the age between these groups should be around 8.12 cmH_2_O. According to the results of this study, the difference in MEP between the high and low pregnancy groups is 18.07 cmH_2_O in the fully adjusted model. This difference is more than twice what would be expected based on the mean age difference alone and shows the strong effect of lifetime pregnancies on this variable. Moreover, the association between MEP and lifetime pregnancies was significant even after statistical adjustment for the participant’s current age.

A previous study showed that small reductions in respiratory muscle strength are enough to accelerate the rate of mobility decline associated with aging [20]; this result signifies the clinical relevance of the large differences observed across the childbearing categories presented in this study’s sample.

This study is particularly valuable because research on the relationship between respiratory muscle strength and reproductive characteristics is lacking from the literature. In a rare exception, researchers examining healthy Tunisian women aged ≥ 45 observed an association between multiparity and lung function, evaluated by spirometry, as well as inspiratory muscle strength [23]. The authors concluded that lung function declines with increasing parity, and this association was attributed to many possible causes associated with multiple childbearing, including cumulative hormonal changes (e.g. cortisol secretion), and anatomical modifications in the thorax and respiratory muscles derived from the repetitive augmentation of the uterus, leading to modification in the muscles’ tension-length relationship, which may make them weaker.

According to our results, there is a clear and strong gradient of lower expiratory muscle strength with higher lifetime pregnancies. Physiologically, this association may be explained by alterations in abdominal muscle biomechanics after multiple childbearing. During pregnancy, it is common for the two sides of the recti abdominal muscle to separate from each other, known as recti diastasis; this separation may result in a decline of abdominal strength post-partum [41]. Additionally, our findings reveal the cumulative effect of multiple lifetime pregnancies on the muscles’ biomechanics may have a possible effect on abdominal recti and other respiratory muscles; the association is strongest in the group with the highest number of lifetime pregnancies.

In addition to abdominal muscle alterations, increased post-partum uterine volume induces a change in the configuration of the chest and respiratory muscles [42]. These changes may accumulate after repeated pregnancies [23], leading to a reduction in respiratory muscle strength.

The associations observed between the number of pregnancies and MIP were not sustained after adjustment for the covariates in the linear regression models. Possibly, the differences in MIP between the groups were not large enough for detection in a relatively small sample. Furthermore, our results showed that women with higher BMI presented higher values for MIP. Some theories support increased respiratory muscle strength in obese individuals, who have an excess of adipose tissue in the thoracic cavity and abdomen, and can be explained by an adaptation to chronic overload that is accompanied by obesity [35]. Potentially, the increased volume of the abdominal area during several pregnancies promotes a similar effect on the inspiratory muscles, leading to reduced loss in MIP over the aging process. More studies are necessary to clarify such associations.

An alternative explanation for our findings may be related to hormonal changes resulting from multiple childbearing across the life course. Multiparity may predispose women to chronic stress [23,43], which is associated with higher levels of cortisol secretion [44]. Considering that elevated levels of cortisol are associated with a reduction in muscle strength [23], it is possible that this hormone is an intermediate factor in the relationship between the number of pregnancies and respiratory muscle strength.

The findings of this study suggest that health professionals, especially physiotherapists and respiratory therapists, should promote respiratory muscle exercises, especially for the expiratory abdominal muscles, among pregnant women from the prenatal to postpartum period. Moreover, to reduce expiratory muscle weakness during or after pregnancies and its associated negative effects over time, specific exercises for these muscles should be prescribed for women across the lifespan, particularly for those diagnosed with recti diastasis. Weaker expiratory muscles are associated with ineffective coughing, impaired intestinal transit, atelectasis and mobility decline, which can lead to adverse health outcomes and poor quality of life [5,9,18,24]. These shortcomings are even more significant for low-income populations, since they are more likely to start childbearing earlier [25,26], tend to have multiple children [18], and have greater losses in muscle function with aging [20,45]. In light of this information, this study highlights the need to consider reproductive variables when assessing the respiratory system, especially in aging populations.

This study has some limitations. The sample may not have been large enough to detect small differences in MIP and MEP values across covariates. Also, the difference in MIP values across pregnancy groups may not have been large enough for detection with this relatively small sample. Moreover, maximal respiratory pressures were measured at only one occasion. It is well known that there is a learning effect within these tests which are very effort-dependent, which is why participants performed the test five times and only the highest values were used in these analyses [10,32]. While our protocol is a common procedure in clinical practice and research [10,32] future studies might consider taking these measures on more than one occasion. Moreover, it has been reported that the use of maximal respiratory pressures maneuvers may be limited because of the difficulty of fitting the mouthpiece for evaluation, especially when considering older people, and that the sniff test may be useful for obtaining additional information on inspiratory pressures [32]. However, we did not use a sniff test in our study, and our trained interviewer did not observe any issues with the test that employed a flanged mouthpiece.

Additionally, participants were recruited through convenience sampling, which may limit the external validity of the study. However, this sample mirrors socioeconomic characteristics (family income and education) of women from Santa Cruz according to the latest census data [30]. Finally, some information was collected by self-report and misclassification bias may occur for those with lower education. Nonetheless, self-reported questionnaires are a typical method employed in health literature [46]. Furthermore, we used objective and validated measures of respiratory muscle strength and bias related to recall of the exposure and covariates measures would most likely be non-differential, as it is unlikely that reporting would differ systematically across MIP/MEP values.

## Conclusion

This study provides evidence that multiple lifetime pregnancies influence respiratory muscle strength, where women with a higher number of pregnancies have lower mean values of maximal respiratory pressures. However, when adjusted for confounding variables, this relationship is only maintained for expiratory muscular strength. The relationship between MEP and this reproductive variable may be explained by biomechanical changes in the respiratory muscles influenced by multiple childbearing. Our findings indicate a need to encourage women from the prenatal to postpartum period to safely exercise their respiratory muscles (mainly the abdominal muscles); this encouragement is critical for decreasing the harmful effects of multiple lifetime pregnancies on respiratory muscles, particularly when combined with the effects of aging.

## Supporting information

S1 File Study dataset. (SAV)
